# Domestic

**Published:** 1841-05-29

**Authors:** 


### MEDICAL EXAMINER.

DEVOTED TO MEDICINE, SURGERY, AND THE COLLATERAL SCIENCES.

No. 22.] PHILADELPHIA, SATURDAY, MAY 29, 1841. [Vol. IV.

### DOMESTIC.

*Esteemed Friend,*—In thy communication on the use of Rhatany in the treatment of fissure of the anus, &c., in number twenty of the Medical Examiner, I observe that thou hast been incidentally led into an error respecting the quantity necessary to saturate with its extractive matter a given quantity of liquid.

The proportions should be

Pulv. Rad. Kramer. ℥iss.
Aq. font. ℥vj.

Put the root into a displacement vessel, or glass funnel, with a cork fitted in the bottom; pour the liquor over it, and macerate for an hour or two; withdraw the cork, and repass the liquid two or three times; then displace with an additional quantity of water until ℥vj. has passed. Prepared in this way each ounce will contain from twenty-five to twenty-eight grains of extract, which I believe is as much of the extractive matter as it will take up.

Respectfully, Thomas Oliver.
*Fifth mo. 20th,* 1841.

---

At a special meeting of the Medical Society, held on Friday, the 21st inst., Dr. B. H. Coates was called to the chair.

Dr. Hays announced the death of Dr. W. P. Dewees; and, seconded by Dr. W. Poyntell Johnston, offered the following resolutions, which were unanimously adopted.

*Resolved,* That the Society has heard with deep regret of the decease of their late much respected fellow-member and former Vice President, Dr. Wm. P. Dewees.

*Resolved,* That Dr. Dewees, by his talents, labour, and honourable course through life, contributed materially to the advancement of our science, and to the elevation of professional character.

*Resolved,* That in testimony of our respect for the deceased, the Society will, in a body, attend his funeral.

*Resolved,* That Dr. H. L. Hodge be requested to prepare and read before the Society a memoir of Dr. Dewees.

*Resolved,* That a committee be appointed to express the feelings of the members to the family of Dr. Dewees, and to communicate to them a copy of the above resolutions.

*Resolved,* That the proceedings of the Society be published.

The chairman appointed Drs. Hodge, Hays, and Huston, to carry the fourth resolution into effect. Samuel L. Hollingsworth, Jr., Rec. Sec.

---

Interments *in the City and Liberties of Philadelphia, from the* 15*th to the* 22*d of May*, 1841.

| Diseases. | Adults. | Children. |
|---|---|---|
| Angina pectoris, | 1 | 0 |
| Abscess of lungs, | 1 | 0 |
| —— hip, | 0 | 1 |
| Aneurism of aorta, | 1 | 0 |
| Apoplexy, | 1 | 0 |
| Amennorrhea, | 0 | 1 |
| Burns, | 0 | 1 |
| Cancer of stom'h, | 1 | 0 |
| Croup, | 0 | 2 |
| Congestion of lungs, | 0 | 2 |
| Cholera morbus, | 1 | 0 |
| Consumption of the lungs, | 15 | 5 |
| Convulsions, | 0 | 4 |
| Diarrhœa, | 0 | 1 |
| Dropsy, | 1 | 0 |
| —— abdominal, | 2 | 0 |
| —— head, | 0 | 6 |
| —— breast, | 1 | 0 |
| Disease of brain, | 0 | 1 |
| —— kidneys, | 1 | 0 |
| Debility, | 1 | 1 |
| Epilepsy, | 1 | 0 |
| Erysipelas, | 1 | 0 |
| Effusion on brain, | 0 | 2 |
| Fever, typhus, | 0 | 1 |
| —— typhoid, | 1 | 0 |
| —— puerperal, | 1 | 0 |
| —— scarlet, | 0 | 2 |
| Inflammation of the brain, | 2 | 0 |
| —— bronchi, | 0 | 2 |
| —— lungs, | 3 | 7 |
| —— stomach, | 1 | 0 |
| —— bowels, | 1 | 1 |
| —— liver, | 0 | 2 |
| | — | — |
| *Carried forward,* | 38 | 42 |

| Diseases. | Adults. | Children. |
|---|---|---|
| *Brought forward,* | 38 | 42 |
| Inflammation of breast, | 0 | 2 |
| —— heart, | 1 | 0 |
| —— peritonæum, | 1 | 0 |
| —— uterus, | 0 | 1 |
| Marasmus, | 0 | 1 |
| Measles, | 0 | 10 |
| Palsy, | 1 | 0 |
| Pleuropneumonia, | 1 | 0 |
| Scrofula, | 0 | 1 |
| Small pox, | 0 | 4 |
| Still-born, | 0 | 3 |
| Suicide, | 1 | 0 |
| Spitting of blood, | 1 | 0 |
| Softn'ng of brain, | 1 | 0 |
| Ulcerated sore throat, | 0 | 3 |
| | — | — |
| Total, 111— | 45 | 66 |

Of the above, there

| | |
|---|---|
| were under 1 year | 22 |
| From 1 to 2 | 20 |
| 2 to 5 | 10 |
| 5 to 10 | 8 |
| 10 to 15 | 3 |
| 15 to 20 | 8 |
| 20 to 30 | 13 |
| 30 to 40 | 12 |
| 40 to 50 | 9 |
| 50 to 60 | 3 |
| 60 to 70 | 5 |
| 70 to 80 | 3 |
| 80 to 90 | 0 |
| 90 to 100 | 0 |
| Total, | 130 |

Of the above there were 9 from the almshouse, 18 people of colour, and two from the country, which are included in the total amount.

*Weekly Report of Interments in the City and County of New York, from the 15th to the 22d of May*, 1841.—Cholera Infantum 1; Consumption 27; Convulsions 8; Croup or Hives 1; Delirium tremens 2; Dropsy in the head 8; Drowned 2; Epilepsy 1; Fever 1; do. scarlet 3; do. typhoid 5; do. puerperal 3; do. remittent 2; Fracture 1; Gout 2; Intemperance 3; Inflammation of brain 11; do. of chest 6; do. of lungs 8; do. of bowels 3; Lock Jaw 1; Marasmus 7; Old age 1; Organic disease of heart 2; Rupture 1; Small Pox 5; Unknown 2.

Ages—Of 1 year and under, 28; between 1 and 2, 3; 2 and 5, 15; 5 and 10, 10; 10 and 20, 7; 20 and 30, 18; 30 and 40, 20; and 40 50, 14; 50 and 60, 4; 60 and 70, 2; 70 and 80 2.

